# Using Budding Yeast to Identify Molecules That Block Cancer Cell ‘Mitotic Slippage’ Only in the Presence of Mitotic Poisons

**DOI:** 10.3390/ijms22157985

**Published:** 2021-07-26

**Authors:** Scott C. Schuyler, Hsin-Yu Chen

**Affiliations:** 1 Department of Biomedical Sciences, College of Medicine, Chang Gung University, Kwei-Shan, Taoyuan 333, Taiwan; hsinyu.chen@mail.cgu.edu.tw; 2 Division of Head and Neck Surgery, Department of Otolaryngology, Chang Gung Memorial Hospital, Kwei-Shan, Taoyuan 333, Taiwan

**Keywords:** *Saccharomyces cerevisiae*, budding yeast, cancer, cell cycle, mitosis, spindle assembly checkpoint, anaphase-promoting complex/cyclosome (APC/C), cell division cycle 20 (Cdc20), mitotic checkpoint complex (MCC)

## Abstract

Research on the budding yeast *Saccharomyces cerevisiae* has yielded fundamental discoveries on highly conserved biological pathways and yeast remains the best-studied eukaryotic cell in the world. Studies on the mitotic cell cycle and the discovery of cell cycle checkpoints in budding yeast has led to a detailed, although incomplete, understanding of eukaryotic cell cycle progression. In multicellular eukaryotic organisms, uncontrolled aberrant cell division is the defining feature of cancer. Some of the most successful classes of anti-cancer chemotherapeutic agents are mitotic poisons. Mitotic poisons are thought to function by inducing a mitotic spindle checkpoint-dependent cell cycle arrest, via the assembly of the highly conserved mitotic checkpoint complex (MCC), leading to apoptosis. Even in the presence of mitotic poisons, some cancer cells continue cell division via ‘mitotic slippage’, which may correlate with a cancer becoming refractory to mitotic poison chemotherapeutic treatments. In this review, knowledge about budding yeast cell cycle control is explored to suggest novel potential drug targets, namely, specific regions in the highly conserved anaphase-promoting complex/cyclosome (APC/C) subunits Apc1 and/or Apc5, and in a specific N-terminal region in the APC/C co-factor cell division cycle 20 (Cdc20), which may yield molecules which block ‘mitotic slippage’ only in the presence of mitotic poisons.

## 1. Introduction

Cancer is a collection of diseases unified by displaying rounds of uncontrolled cell division [1]. Chemotherapy has been a partially successful approach to attack cancer cells for decades, wherein one of the primary classes of chemical agents are the mitotic poisons. Prominent among mitotic poisons are microtubule poisons that alter microtubule function [2]. Cells respond to the presence of microtubule poisons, in part, by inducing a cell cycle arrest in mitosis that depends upon a pathway called the spindle assembly checkpoint [3]. Cell cycle progression is governed by rounds of synthesis, activation, and destruction of cyclins, which are activators of cyclin-dependent kinases, as well as by the production and destruction of other critical factors. The destruction of cyclins and other cell cycle regulated factors occurs via ubiquitin-dependent proteolysis, where the core E3-ubiquitin ligase in mitosis is the anaphase-promoting complex/cyclosome (APC/C) [4]. The activation of this enzyme requires a physical interaction with the essential mitotic co-factor cell division cycle 20 (Cdc20), which recruits target substrates to the holoenzyme to promote their poly-ubiquitinylation [5].

**Figure 1:**
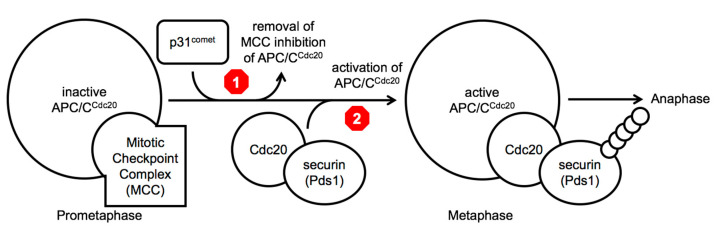
Sequential activation of APC/C^Cdc20^ from prometaphase to anaphase. During each cell cycle, and in the presence of anti-microtubule mitotic poisons, APC/C^Cdc20^ is inactive in prometaphase. Upon achievement of proper microtubule attachments to kinetochores, MCC inhibition of APC/C^Cdc20^ is removed concurrently with the activation of the APC/C^Cdc20^ enzyme to promote the poly-ubiquitinylation (small circles) of target substrates in metaphase that allows cell cycle progression into anaphase. In some cancer cells, even in the presence of a mitotic poison, the cells progress into anaphase and undergo ‘mitotic slippage’. To block these events, one potential target for inhibition is p31^comet^ (red stop sign 1) to block the removal of MCC inhibition and a second potential target is inhibiting the activation of APC/C^Cdc20^ towards substrates that promote entry into anaphase (red stop sign 2).

In each round of mitosis, as cells enter M-phase, the spindle assembly checkpoint—often referred to as the mitotic spindle checkpoint—delays cell cycle progression until all chromosomes are connected to the mitotic spindle microtubules properly [3,6,7]. The mitotic spindle checkpoint is thought to function by recruitment of the mitotic arrest deficient 1–mitotic deficient arrest 2 (Mad1–Mad2) complex to unattached kinetochores that promotes the conversion of the ‘open’ form of Mad2 into the ‘closed’ form as it creates a ‘safety-belt’ binding loop around the N-terminal ‘Mad2-binding motif’ of Cdc20 [3,6,7]. This Mad2–Cdc20 complex then is proposed to interact with the APC/C pseudo-substrate Mad3 along with its binding partner, budding uninhibited by benomyl 3 (Bub3), to form the mitotic checkpoint complex (MCC) [3,6,7]. The sequestration of the essential APC/C co-factor Cdc20 within the MCC is proposed to block the activation of APC/C^Cdc20^, thus blocking cell cycle progression in prophase and prometaphase of mitosis (Figure 1). During this cell cycle arrest, the p31^comet^ protein is thought to promote the release of Cdc20 from Mad2 promoting the disassembly of the MCC and the destruction of the MCC-bound Cdc20 protein via APC/C^Cdc20^ ubiquitin-dependent proteolysis [3,6,7]. Once all kinetochores are attached properly to the mitotic spindle via microtubules, the conversion of ‘open’ Mad2 to the ‘closed’ form ends, leaving newly produced Cdc20 to bind to the APC/C and to target substrates, such as securin (precocious dissociation of sisters 1 (Pds1) in budding yeast), promoting their poly-ubiquitinylation and subsequent destruction by the 26 proteosome and allowing entry into anaphase [3,6,7]. 

Anti-cancer mitotic poisons, such as paclitaxel (Taxol), are thought to function by preventing proper kinetochore–microtubule interactions, inducing an extended mitotic checkpoint arrest, which leads cells to execute apoptosis, a form of genetically programmed cell death [8]. However, even in the presence of mitotic poisons, some cancer cells display the ability to execute an alternative process, and instead of executing apoptosis, they advance in the cell cycle into anaphase via a process called ‘mitotic slippage’ [8,9,10,11,12]. The resultant daughter cells from such a ‘mitotic slippage’ event very frequently die, presumably because of the massive levels of chromosome mis-segregation, chromosome loss, and chromosomal double-strand breaks that all occurred during the previous aberrant mitotic segregation when the microtubules of the spindle were not functioning properly. However, in cancers, even if only a very small number of these daughter cells survive, it appears to be enough to allow the tumors to continue to grow. Thus, a hypothesis was formed that one potential avenue to explore for the creation of a mitotic poison combination chemotherapy agent would be the discovery of a small molecule(s) that could extend the mitotic delay induced by a mitotic poison and prevent ‘mitotic slippage’.

Two different molecular steps in the same pathway have been investigated as leading candidates to target to extend mitotic poison dependent-arrests, namely to either block the inactivation of the MCC (Figure 1 above, red stop sign 1), creating a longer mitotic arrest, or to block the activity of the APC/C^Cdc20^ holoenzyme, preventing the ubiquitinylation of metaphase target substrates (Figure 1 above, red stop sign 2) [8,9,10,11,12,13,14,15]. In lab experiments working with a variety of different cancer cells lines, both approaches have been validated in proof-of-principle experiments making use of RNAi techniques to remove the targeted proteins involved in each step. In 2010 and 2011, a decrease in p31^comet^ was observed to extend a mitotic arrest, and more recently in 2021, it was demonstrated that removal of p31^comet^ by RNAi leads to increased sensitivity to mitotic poisons and increased levels of apoptosis [11,12,13,14,15]. At the same time, around 2009–2011, others demonstrated that the removal of Cdc20, again by RNAi, led to increased apoptosis in the presence of mitotic poisons [8,9,10]. Although RNAi is currently not a viable therapeutic technology, this collection of results demonstrates that inhibition of p31^comet^ or inhibition of APC/C^Cdc20^ by depleting Cdc20 could extend the spindle checkpoint delay induced by mitotic poisons forcing cancer cells to undergo apoptosis and not allow ‘mitotic slippage’.

Thus, the search began in earnest to identify small molecules that might block p31^comet^ or APC/C^Cdc20^ function. To date, no p31^comet^ inhibitor has been reported, perhaps in part because it is not clear which part of the molecule to target, although detailed crystallographic and other studies have provided insights into important regions and amino acid motifs that appear to be critical for its function, which may serve as promising targets, perhaps by targeting these regions with small stable proteomimetic peptides to try and disrupt critical interactions [4]. There has been more success in the discovery of several APC/C inhibitors, where notably one of them called apcin targets Cdc20 directly [16,17,18,19]. This APC/C Cdc20 co-factor specificity is critical because the APC/C has other co-factors involved in promoting cell cycle progression into and throughout G1, namely, CDC20 homolog 1 (Cdh1) [20,21]. Furthermore, the APC/C is known to have other well-established functions in terminally differentiated cells, such as neurons, creating concerns for any novel therapeutic agent about off-target effects [22]. However, the current only known essential function of the APC/C^Cdc20^ form of the holoenzyme in humans is the promotion of somatic cell mitotic cell division, or it is also likely to be involved in the promotion of the metaphase II–anaphase II advance during meiosis II in gametogenesis, as in mice [23]. Surprisingly, even the most promising APC/C^Cdc20^ inhibitor molecule apcin gave an unexpected result, namely, in the absence of mitotic poisons, it induced the expected cell cycle arrest at metaphase, but in the presence of mitotic poisons, which would be the expected conditions in cancer cells in individuals undergoing chemotherapy, apcin induced ‘mitotic slippage’, rather than preventing it, by not only decreasing APC/C^Cdc20^ activity, but also by blocking mitotic checkpoint MCC function, the opposite of the desired result [24]. Thus, the goal to identify a specific APC/C^Cdc20^ inhibitor that can extend a mitotic poison induced spindle checkpoint arrest remains unfulfilled.

With this unfulfilled goal in mind, outlined below is a summary based on knowledge of the budding yeast cell cycle in mitosis, which leads to the suggestion that two highly conserved APC/C subunits, namely, Apc1 and Apc5, and a specific N-terminal-interacting region in the co-factor Cdc20, in between the Cdc20 ‘C-box’ and ‘KILR’ motifs, are novel potential target sites to inhibit APC/C^Cdc20^ function in a manner that may satisfy the current goals in the field to identify small molecules that (i) only cause an extended mitotic arrest in the presence of a mitotic poison to prevent cancer cell ‘mitotic slippage’, (ii) do not affect MCC function, and (iii) specifically inhibit APC/C^Cdc20^ and not APC^Cdh1^ function.

## 2. Target Sites for Potentially Inhibiting APC/C^Cdc20^ Function

From the proof-of-principle work on human cancer tissue cells, p31^comet^ and APC/C^Cdc20^ are currently suggested to be the two best candidates to try and target, wherein it has been shown by RNAi that removing p31^comet^ or Cdc20 function in the presence of a mitotic poison will greatly increase the promotion of apoptosis and block or decrease ‘mitotic slippage’ [8,9,10,11,12,13,14,15]. In budding yeast, the best potential current candidate for a p31^comet^ homolog is tiny yeast comet 1 (Tyc1) [19]. Although this protein was identified based on its homology with p31^comet^, and its ability to interact with Mad2, it remains unclear to what extent it is a functional homolog of p31^comet^. Thus, here, the focus is on exploring past budding yeast phenotypic knowledge about APC/C^Cdc20^, which is well established as a true functional homolog of human APC/C^Cdc20^ activity in catalyzing the metaphase-anaphase transition, and as the target of the MCC to induce a cell cycle arrest in the presence of mitotic poisons, in order to potentially identify proteins, domains, and amino acid motifs to target during novel drug screens. 

First, a comparative list of the protein subunits and co-factor of the human and budding yeast APC/C^Cdc20^ enzymes are shown (Table 1) [4]. A substantial degree of similarity is displayed, even though potentially more than 1 billion years of evolution is thought to separate humans and budding yeast. The Apc7 and Apc16 subunits in the human holoenzyme appear to be absent in yeast, where Apc7 is a TPR protein and the function of the very small molecular weight Apc16 protein is unknown [4]. Conversely, the Apc9 subunit is present in the budding yeast holoenzyme, but absent in the human complex, where the function of yeast Apc9 is unknown. 

Second, among the APC/C^Cdc20^ proteins, past phenotypic data were explored to look for a specific unusual phenotype: an *increased* resistance to a mitotic poison in the mutant cells, where the typical one employed to activate the mitotic spindle checkpoint in yeast is benomyl, which promotes microtubule depolymerization. The reasoning behind a search for this type of very unique *increased* resistance to a mitotic poison phenotype was as follows: yeast cells do not develop cancer, and whether they undergo a type of apoptosis is a matter of debate. High levels of benomyl kill wild-type cells, where the only known essential function of microtubules during vegetative growth in budding yeast is chromosome segregation in mitosis [27]. Presumably, the benomyl-induced cell death correlates with loss of microtubule function in mitosis, where at high levels of the molecule, the cells cannot recover mitotic spindle function, and presumably die from an inability to segregate their chromosomes properly. Yeast cells are reported have mechanisms to try and lower the amount of benomyl, such as by pumping the compound into the vacuole, pumping compounds back outside of the cell, and/or metabolizing them into a form that no longer promotes microtubule depolymerization [28]. Thus, any mutation in an APC/C subunit that increases the amount of time in mitosis might lead to an increase in the amount of time a cell has to pump and/or process the benomyl, allowing the cell to recover enough tubulin function to successfully construct a functional mitotic spindle that allows for successful chromosome segregation, as well as the creation of viable daughter cells, as indicated by yeast colony growth on plates. There are many such mutants that display an increased resistance to benomyl in yeast cells, but, notably, only one of them has been identified among the subunits in the APC/C^Cdc20^, namely, Apc5 (Table 1) [25,26]. This is also striking because an Apc5 mutant allele has been isolated and characterized in the nematode *C. elegans*, wherein the mutation causes an extended mitotic arrest that depends upon the mitotic spindle checkpoint, suggesting blocking Apc5 function may block mitotic exit by extending a mitotic checkpoint arrest [29]. Combined, these observations suggest that blocking Apc5 function in an APC/C^Cdc20^ holoenzyme may extend a mitotic arrest in the presence of a mitotic poison.

**Figure 2:**
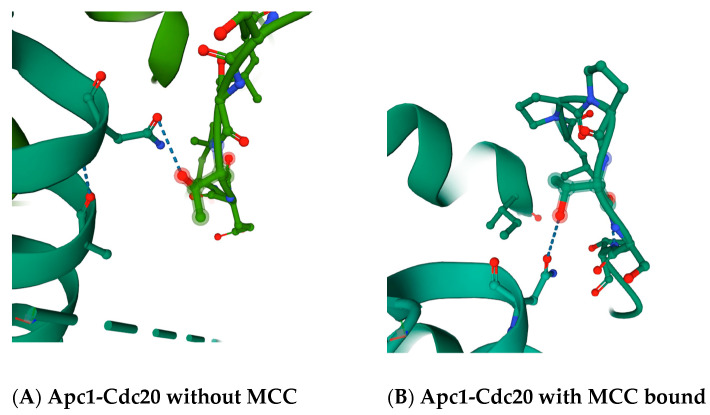
Proposed interaction sites between amino acid residues in human Apc1 and human Cdc20. (**A**) The modeled interactions in the absence of the mitotic checkpoint complex (MCC) between threonine T1239 in Apc1 (displayed in light green) with asparagine N119 in Cdc20 (displayed in dark green). Dashed blue-gray lines denote modeled interactions. (**B**) The modeled interactions in the presence of the mitotic checkpoint complex (MCC) between threonine T1241 in Apc1 (displayed in green on the right) with asparagine N119 in Cdc20 (shown in green on the left). Dashed blue-gray lines denote modeled interactions. Images are from the RCSB PDB (rcsb.org) of PDB ID 6Q6G (without MCC) and PDB ID 6TLJ (with MCC bound) [4,31,32].

**Figure 3:**
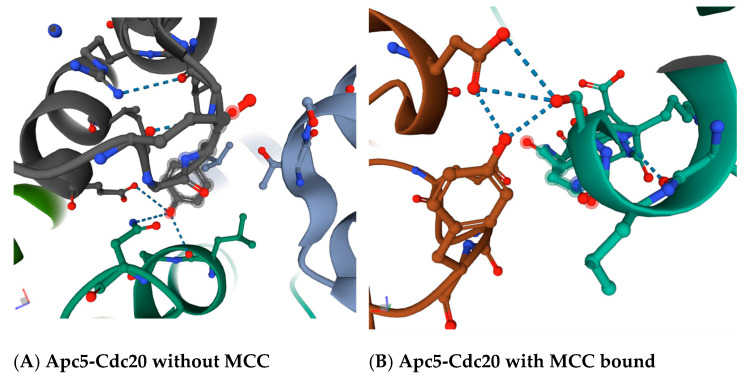
Proposed interaction sites between amino acid residues in human Apc5 and human Cdc20. (**A**) The modeled interactions in the absence of the mitotic checkpoint complex (MCC) between tyrosine Y296 in Apc5 (displayed in charcoal gray) with leucine L95 and asparagine N99 in Cdc20 (displayed in dark green). Dashed blue-gray lines denote modeled interactions. (**B**) The modeled interactions in the presence of the mitotic checkpoint complex (MCC) between tyrosine Y296 in Apc5 (displayed in brown) and glutamic acid E333 in Apc5 (displayed in brown) with serine S96 in Cdc20 (shown in dark green). Dashed blue-gray lines denote modeled interactions. Images are from the RCSB PDB (rcsb.org) of PDB ID 6Q6G (without MCC) and PDB ID 6TLJ (with MCC bound) [4,31,32].

An interest in this very unique Apc5 phenotype of increased resistance to benomyl is buttressed by the fact that the N-terminus of the Cdc20 co-factor contains a loop that interacts with Apc1 and Apc5 in between the essential APC/C activation motif called the ‘C-box’ and a second APC/C activation motif called the ‘KILR motif’ [4]. The ‘C-box’ of Cdc20 interacts with the Cdc23/Apc8 APC/C subunit, and this interaction is essential for APC/C^Cdc20^ activity. The ‘KILR motif’ of Cdc20 interacts with the Apc8/Cdc23 subunit in APC/C^Cdc20^, and this interaction is also essential for activity [30]. The ‘KILR motif’ is also the direct target of the mitotic spindle checkpoint, where the location where Mad2 binds and inhibits Cdc20 overlaps directly with all of the ‘KILR motif’ amino acid residues, where the ‘Mad2-binding motif’ extends both towards the N- and C-termini of Cdc20 around the ‘KILR motif’ [30]. The region in between the ‘C-box’ and the ‘Mad2-binding motif’, which includes the ‘KILR motif’, forms a loop that in a variety of crystal structures makes potential direct contacts with the Apc5 subunit, and also loops around to make a potential direct contact with the Apc1 subunit (Figure 2 and Figure 3). The proposed contact between the human Cdc20 amino acid residue asparagine N119 is with the threonine T1239 residue in Apc1 in the absence of MCC binding (Figure 2A), and between asparagine N119 in Cdc20 and the threonine T1241 in Apc1 in the presence of MCC binding (Figure 2B). The proposed contact between the leucine L95 and asparagine N99 in Cdc20 is with tyrosine Y296 in Apc5 in the absence of MCC binding (Figure 3A), and between serine S96 in Cdc20 with both the tyrosine Y296 and the glutamic acid E333 in Apc5 in the presence of MCC binding (Figure 3B). These potential contacts have been proposed to exist in several different modeled human APC/C^Cdc20^ structures, increasing the probability they are real, and are absent in a model of the APC/C^Cdh1^ holoenzyme, indicating they may be unique to APC/C^Cdc20^ holoenzymes [4].

**Figure 4:**
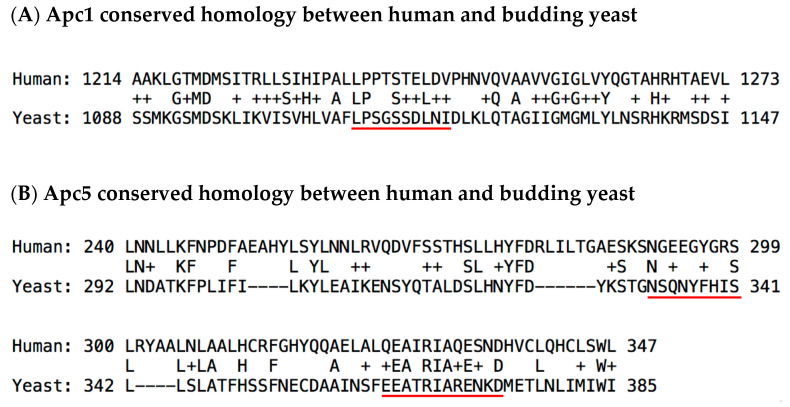
Levels of conservation of amino acids modeled as displaying interactions between human Cdc20-Apc5 and human Cdc20-Apc1. (**A**) In Apc1, the core potential interacting region is at the amino acid motif 1236-LPPTSTLDV-1244, which corresponds to the budding yeast amino acid motif of 1110-LPSGSSDLNI-1119 (red underline). In human Apc1, there is a cluster of serine and two threonines that display the modeled interactions, which corresponds to cluster of three serines in yeast Apc1. The structural similarity between serine and threonine may be important for the interaction with asparagine N119 from Cdc20. (**B**) In APC/C without MCC bound, the Apc5 core potential regions are at the amino acid motifs of 291-NGEEGYGRS-299, which corresponds to the budding yeast amino acid motif of 333-NSQNYFHIS-341 (red underline). Although the alignment places the human tyrosine Y296 together with phenylalanine F338, it should be noted there is an adjacent tyrosine at Y337 that may be involved in any potential interaction with Cdc20 in yeast. In APC/C with MCC bound, in addition to the modeled interaction with tyrosine Y296, there is a seconded modeled interaction with glutamic acid E333, where the core human amino acid motif is 325-QEAIRIAQESND-336, which corresponds to the budding yeast amino acid motif of 363-EEATRIARENKD-374 (red underline).

Although it is intriguing that Apc5 has the notable unique phenotype of being resistant to a mitotic poison in yeast and induces a mitotic checkpoint-dependent extended delay in *C. elegans*, these results become more compelling when the potential physical interactions between Cdc20 and Apc5 are explored. Unfortunately, there is no high-resolution ultra-structure reported for the complete budding yeast APC/C^Cdc20^ holoenzyme. However, there is a lower resolution structure based on cryo-electron microscopy analyses demonstrating that the overall organization and architectural shape of the holoenzyme is conserved [33]. In addition, a high degree of similarity has been established between both the human and yeast Apc4 and Apc5 [34,35]. Thus, if the most important contact amino acid residues in Apc1 and Apc5 are highly conserved from yeast to humans, and the overall architecture of the APC/C is conserved, then there is a reasonable chance that the proposed direct contact interactions between the N-terminus of Cdc20 with Apc1 and Apc5 may also be conserved. To investigate this possibility, a series of basic local alignment search tool (BLAST) analyses were performed to investigate if the Apc1 threonine amino acid residues T1239 and T1241 are conserved from yeast to humans, along with the Apc5 amino acid residue tyrosine Y296 and glutamic acid E333 (Figure 4). BLAST alignments show compelling evidence to indicate that the Apc1 and Apc5 critical potential Cdc20-interacting residues are partially or highly conserved, and thus may serve as target sites for future small molecule screens to identify APC/C^Cdc20^ inhibitors. It is also known from previous work that a 29 amino acid peptide derived from budding yeast Cdc20 in the region between the ‘C-box’ and the ‘Mad2-binding motif’ can function as an APC/C^Cdc20^ inhibitor in vitro [19].

## 3. Discussion

Going forward, in order to create a novel potential mitotic poison combinatorial anti-cancer therapeutic molecule, the goal in the field is to identify a small molecule that (i) only causes an extended mitotic arrest in the presence of a mitotic poison to prevent cancer cell ‘mitotic slippage’; (ii) does not affect MCC function; and (iii) specifically inhibits APC/C^Cdc20^ and not APC^Cdh1^, where the question remains: how can budding yeast help? From past results, it has been modeled that there is a loop in the Cdc20 protein in between the ‘C-box’ and the ‘Mad2-binding motif’ or ‘KILR motif’ that interacts with Apc1 and Apc5 at highly conserved residues [4,31,32]. In addition, an Apc5 mutation displays the very rare and unique phenotype among the APC/C subunits of displaying an increased resistance to the mitotic poison benomyl and can also induce an extended mitotic arrest that depends upon the mitotic spindle checkpoint [25,26,29]. Finally, a 29 amino acid portion of Cdc20 derived from this loop region in between the ‘C-box’ and the ‘Mad2-binding’ or ‘KILR motif’ has been observed to be an APC/C^Cdc20^ inhibitor [19]. At this point, this budding yeast knowledge remains intriguing rather than compelling. What additional studies should be performed to determine if Cdc20-interacting residues of Apc1 and/or Apc5, as well as the surrounding conserved amino acid motifs, might serve as promising targets to achieve the above goals required of a novel mitotic poison combinatorial anti-cancer therapeutic molecule?

First, a test may be performed in budding yeast to determine if the over expression of the region derived from Cdc20 from between the ‘C-box’ and the ‘Mad2-binding motif’ or ‘KILR motif’ does not affect cell growth in the absence of benomyl, and then also enhances cells growth in the presence of benomyl. If cell growth is unaffected in the absence of benomyl, this indicates that APC/C^Cdc20^ and APC/C^Cdh1^ functions remain intact, as both are required for normal cell cycle progression and cell growth. However, if in the presence of benomyl the over expression of the peptide displays an *increased* resistance to benomyl, it would indicate that, specifically during a mitotic poison-induced arrest, the peptide can block APC/C^Cdc20^ function in a manner that extends the arrest time, which is precisely the desired molecular feature of a potential therapeutic molecule. The region between the yeast Cdc20 ‘C-box’ and ‘Mad2-binding motif’ or ‘KILR motif’ is about 52 amino acids long, which means a peptide of this length, even if it displayed the desired phenotypes, would not have any potential therapeutic value simply because it would be too big to enter a cell. However, this set of phenotypes would strongly reinforce the hypothesis that targeting the interactions between Apc1 and/or Apc5 and the Cdc20 protein in this specific loop regions does have promising potential, because they would satisfy the first and third criteria stated above: that the mechanism of action of the small molecule only causes an extended mitotic arrest in the presence of a mitotic poison to prevent cancer cell ‘mitotic slippage’, and that it specifically inhibits APC/C^Cdc20^ and not APC^Cdh1^.

Second, it would be necessary to perform an experiment to show, for example, that the same over expressed peptide derived from Cdc20 from between the ‘C-box’ and the ‘Mad2-binding motif’ or ‘KILR motif’ does not affect MCC function by observing under the light microscope if any cells escape from a benomyl arrest when the peptide is expressed. This experiment is required because of the unexpected result displayed by apcin, where it displayed great promise as an APC/C^Cdc20^-specific inhibitor, but, unfortunately, also disrupts the function of the MCC, thus allowing cancer cells to still execute ‘mitotic slippage’ by entering anaphase, even in the presence of a mitotic poison [24]. 

If these two criteria can be met by the over expression of the Cdc20-derived peptide, then it greatly increases the promise that targeting Apc1 and/or Apc5 highly conserved sites that interact with Cdc20. Thus, budding yeast could then be employed to create functional screens to search for small molecules with the following properties: (i) the ability to disrupt or modify the strength of the Apc1–Cdc20 interaction, (ii) the ability to disrupt or modify the strength of the Apc5–Cdc20 interaction, and/or (iii) the ability to increase the resistance to benomyl in a growth assay by targeting the Apc1–Cdc20 and/or Apc5–Cdc20 interaction.

Towards this end, many well-established technologies and tools in budding yeast could be employed to accelerate the rate of drug discovery. First, site-directed mutagenesis combined with homologous recombination genomic integrates could be performed to create unique mutant alleles at the *apc1*, *apc5*, and *cdc20* loci, replacing single amino acids with alanine to remove their function, such as yeast serine residue S1114 in Apc1 or yeast tyrosine residue Y337 and/or glutamic acid residue E371 in Apc5, or by replacing several conserved amino acids that may serve as a functional motif, such as yeast Apc1 1110-LPSGSSDLNI-1119 and yeast Apc5 333-NSQNYFHIS-341 and/or 363-EEATRIARENKD-374 residues, and further investigating these unique constructed alleles for their displayed level of resistance to benomyl, or their ability to delay cell cycle progression during a benomyl-induced, MCC-dependent arrest. These unique alleles may also be employed during sub-cloning of fragments of the Apc1, Apc5, and Cdc20 proteins into yeast two-hybrid vectors where the goal would be to have a set of positive interactions using native wild-type amino acid residues, and a potential set of negative controls where the interactions have been weakened or destroyed. These two hybrid constructs could then be employed to screen chemical or natural product libraries for molecules that disrupt the interaction, where then a genetic selection can be created such that if the interaction occurs the yeast cells die. Although in vivo screening is preferred, as the native cellular context provides critical functions in all signaling pathways, the molecular interactions outlined here may also be recreated in vitro with pure recombinant proteins and the precisely defined one-on-one physical interactions can by studied in a purely defined system too.

Although there is optimism about employing budding yeast in this way to accelerate potential drug discovery in a rational way, there are likely to be a great many challenges in pursuit of the outlined goals. One major risk is that the crystal structure of the budding yeast APC/C^Cdc20^ holoenzyme is not known at the same level of resolution as the human APC/C^Cdc20^ complex. Although the overall structure of human APC/C and budding APC/C are very similar, it remains unknown if the specific amino acid residues interact in the precise same manner from yeast to humans, although this may be likely based on structural conservation arguments [4,31,32]. However, the demonstrated conservation of the proposed interacting amino acid residues gives one hope that such precision has been maintained during evolution. A second challenge may be presented by the nature of the mitotic poison employed during experiments. In the work highlighted above, benomyl, which depolymerizes microtubules, was employed because it is cheap and easy to work with. However, the most commonly used mitotic poison used in anti-cancer therapy is paclitaxel (Taxol), which stabilizes microtubules. Thus, it may be preferred to investigate phenotypes of mutant alleles in the presence of Taxol rather than benomyl. Native budding yeast are not sensitive to Taxol because it does not effectively traverse the cell wall and plasma membranes of yeast cells, and also because the Taxol binding site is not present on yeast microtubules. However, a system was recently successfully developed that allows for the use of Taxol in budding yeast, which may be necessary to work with when screening for small molecules that may function in combination with a Taxol-induced mitotic arrest [36]. Finally, it remains an open debate if the proper way to attack cancer cells is by extending a mitotic poison-induced arrest, or, alternatively, if a better strategy is to try and destroy spindle checkpoint function and force cancers cells to exit mitosis pre-maturely in the presence of a mitotic poison, leading to catastrophic levels of chromosome mis-segregation that even cancer cells cannot tolerate. The argument for this approach has recently been buttressed by the observation that as cancer cells become more and more aneuploid, which tends to correlate with the stage and aggressiveness of many cancers, they also become more and more sensitive to the removal of mitotic spindle checkpoint function [37]. Thus, rather than enhancing the checkpoint-dependent arrest, a better strategy may be to disrupt the mitotic spindle checkpoint to make the cell cycle delay as short as possible. Or, both approaches may be valid, where in early stage cancers it may be better to attack the cancer cells with combined therapies that force them to undergo an extended mitotic delay increasing the rate of apoptosis, but in later stage cancers, it may be better to attack cancers cells with agents that remove spindle checkpoint function. Further work in this domain may be necessary in order to clarify these uncertainties.

Between a strategy to delay cell cycle progression and a strategy to accelerate cell cycle progression in the presence of a mitotic poison, we are in agreement with the argument as outlined recently by Henriques et. al. (2019) and prefer the concept of trying to block mitotic exit completely, via ‘mitotic slippage’ or any other mechanism (Figure 1) [14]. This preference stems from concerns about the striking resilience of cancer cells to tolerate massive levels of chromosomal rearrangements and other forms of extraordinary high levels of cellular stress. The concern is that if cancers cells are provided any opportunity to exit from mitosis, even if overwhelmingly those cellular divisions are catastrophic and lethal events, it still provides the cells a very small, but non-zero chance to continue to grow and divide. If a compound can be identified that completely prevents cell cycle progression in combination with mitotic poisons, then this would eliminate the ability of cancer cells to ‘roll the dice’ and exit mitosis, potentially giving rise to a viable cell that is capable of executing further cell divisions successfully and allowing the cancer to progress and to continue to evolve into a more aggressive and lethal form.

In conclusion, even with the great potential challenges and uncertainties outlined above, we argue that the deep knowledge about how cell cycle signaling pathways work to make the critical decisions during cell cycle progression will be of great use as the yeast cell cycle field moves forward to try and identify small molecules that are needed to enhance the ability of mitotic poisons to disrupt cell cycle progression in cancer cells in a manner that induces cancer cell death. The collection of detailed knowledge and plethora of molecular and cellular tools and phenotypes has placed budding yeast in a strong position as a model system moving forward to be a useful tool for further identification and characterization of specific molecular targets, such as the modeled potential Apc1-Cdc20 and/or Apc5–Cdc20 interaction sites, for drug discovery. The hope is that this knowledge and the large number of molecular tools in budding yeast can be employed to rapidly identify molecules that only cause an extended mitotic arrest in the presence of a mitotic poison without affecting MCC function by specifically only inhibiting APC/C^Cdc20^.

## 4. Materials and Methods

Protein names and sequences were derived from the National Center for Biotechnology Information (NCBI) (https://www.ncbi.nlm.nih.gov/, accessed on 27 June 2021) database for human proteins and the Saccharomyces Genome Database (SGD) (http://www.yeastgenome.org/, accessed on 27 June 2021) for yeast proteins [38,39,40]. Information about budding yeast gene phenotypes were obtained from SGD and The Cell Map (https://thecellmap.org/, accessed on 27 June 2021) databases [38,41,42,43]. Crystal structure analyses were performed using the Research Collaboratory for Structural Bioinformatics Protein Data Bank (RCSB PDB) (https://www.rcsb.org/, accessed on 27 June 2021) [31,32]. Protein–protein homology searches and amino acid sequence alignments were performed using the basic local alignment search tool (BLAST) at the NCBI and SGD databases [38,39,40].

## Figures and Tables

**Table 1:** APC/C^Cdc20^ subunits from human and budding yeast. Only a mutation in yeast Apc5 is known to display the very unique phenotype of an increased resistance to a mitotic poison (denoted as a “+” sign) [25,26].

Human	Budding Yeast	Yeast Mutant Has Increased Resistance to Mitotic Poison
Apc1	Apc1	−
Apc2	Apc2	−
Apc6	Cdc16	−
Apc3	Cdc27	−
Apc7	−	−
Apc5	Apc5	+
Apc4	Apc4	−
Apc8	Cdc23	−
Apc15	Mnd2	−
-	Apc9	−
Apc10	Doc1	−
Apc11	Apc11	−
Apc12	Cdc26	−
Apc13	Swm1	−
Apc16	−	−
Cdc20	Cdc20	−
